# Mystery Behind a Broken, Discolored Right Maxillary Central Incisor: A Case Report

**DOI:** 10.7759/cureus.22828

**Published:** 2022-03-03

**Authors:** Ravikumar Pethagounder Thangavelu, Karthik Rajaram Mohan, Saramma Mathew Fenn

**Affiliations:** 1 Oral Medicine and Radiology, Vinayaka Mission's Sankarachariyar Dental College, Vinayaka Mission's Research Foundation, Salem, IND

**Keywords:** odontoma, non-vital tooth, mesiodens, infection, hamartoma

## Abstract

Odontoma is a hamartomatous benign odontogenic tumor that may resemble tooth-like structures. Odontomas can impede the eruption of the permanent underlying tooth or can cause devitalization of the tooth resulting in pain and swelling near the tooth, causing diagnostic dilemmas to the dentist. We describe a case of an unusual occurrence of such an odontoma in a 31-year-old male patient in the periapical region of a broken, discolored maxillary central incisor tooth causing a diagnostic dilemma and its treatment.

## Introduction

Odontoma is a hamartomatous, non-neoplastic, non-aggressive odontogenic tumor. Odontoma that resembles a tooth-like structure is called a compound odontoma. Those that do not mimic a tooth-like structure, comprising irregular calcified mass called denticle, are called complex odontomas. Odontomas are typically incidentally noted on routine radiographic examinations when radiographs are taken for other complaints such as a missing tooth.

The clinical signs and symptoms vary with the location and size of the odontoma. When an odontoma occurs inside the alveolar bone, it can impede the eruption or cause deflection of the underlying permanent tooth. When it occurs near the tooth, it can cause tooth malformations, cystic degeneration resulting in bone resorption, and loss of pulp vitality of the adjacent tooth, which results in pain and swelling, causing facial asymmetry.

Smaller-sized odontoma can occur in the mastoid region near the ear in an extragnathic location and is called extragnathic odontoma; it is clinically asymptomatic and does not cause any pain, facial asymmetry, or discomfort. A larger odontoma can lead to transient diplopia if it invades the orbital floor. An odontoma can also mimic symptoms of chronic maxillary sinusitis, such as nasal congestion if it involves the maxillary sinus, causing diagnostic dilemmas and often challenging for the dentist.

## Case presentation

A 31-year-old male patient reported to our oral medicine and radiology department with a chief complaint of pain and swelling in the upper front tooth region for the past two months. On eliciting a history, the patient revealed a history of trauma to his upper front tooth region 6 months back by accidentally slipping down while climbing the stairs, following which his upper front tooth got broken and pain started in the broken upper front tooth region, which was throbbing and non-radiating in character. Pain followed by an intraoral swelling near the labial aspect of the maxillary anterior gingiva was noted.

**Figure 1:**
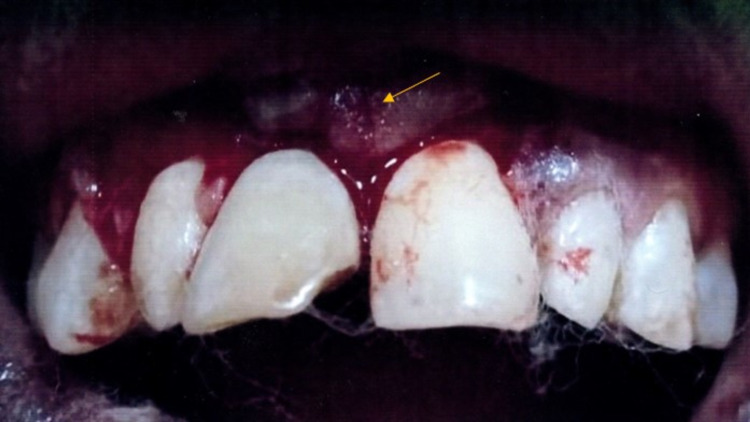
Intraoral clinical photograph revealing the broken right maxillary central incisor #8 associated with a sinus (pin-point) opening

Intraoral clinical examination revealed a loss of mesio-incisal crown portion of #8 (International Tooth Numbering system) and discoloration due to fracture caused by trauma. An intraoral swelling was present in relation to the labial aspect of maxillary attached gingiva in relation to #8. A pinpoint (sinus) opening is present near the mucogingival junction in relation to fractured tooth 11 (Figure 1).

On palpation, pus discharge was present due to intraoral swelling in the maxillary labial vestibule and labial aspect of the attached gingiva in #8. The intraoral swelling was soft in consistency and tender and fluctuant on palpation. Based on the clinical findings, a provisional diagnosis of chronic periapical abscess in the non-vital right maxillary central incisor with intraoral sinus was made.

**Figure 2:**
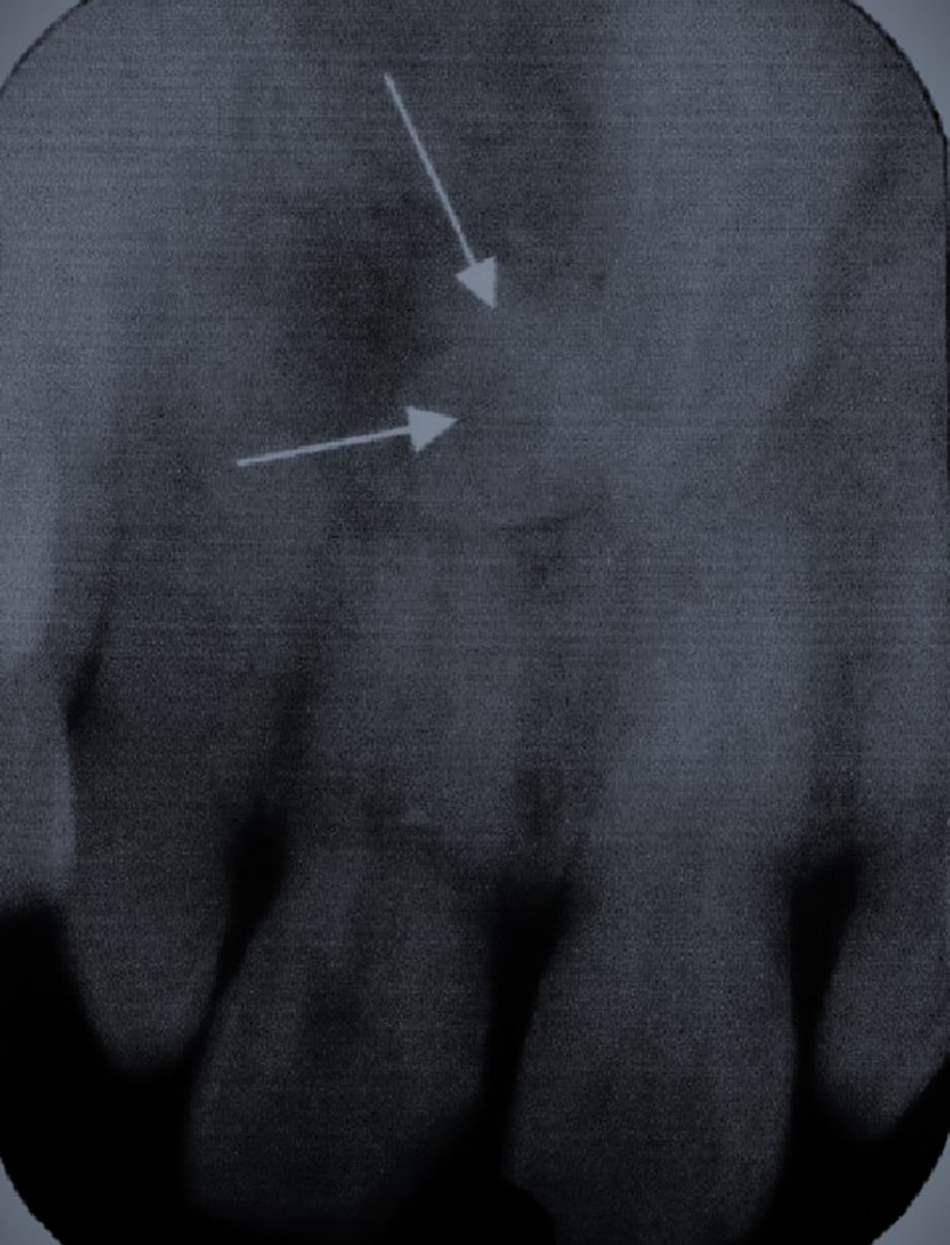
Intraoral periapical radiograph revealing a donut-like radiopacity (indicated by white arrow mark) in the periapical root region of the broken right maxillary central incisor tooth

The intraoral periapical radiograph revealed discrete, well-defined radiopacity measuring approximately 1 cm in diameter near the periapical region of the anterior maxillary teeth bordered by a peripheral rim of radiolucency (Figure 2).

The radiographic differential diagnoses considered are impacted supernumerary tooth mesiodens, condensing osteitis, cementoblastoma, periapical cemento-osseous dysplasia, and idiopathic osteosclerosis.

Cementoblastomas usually obscure the outline of the root. Periapical cemento-osseous dysplasia in early stages occurs as multiple periapical radiolucencies involving the mandibular anterior teeth regions, and later mature stages appear as multiple periapical radiopacities involving the mandibular anterior teeth. Idiopathic osteosclerosis occurs in radiograph as discrete radiopacities without any history of trauma or carious tooth.

**Figure 3:**
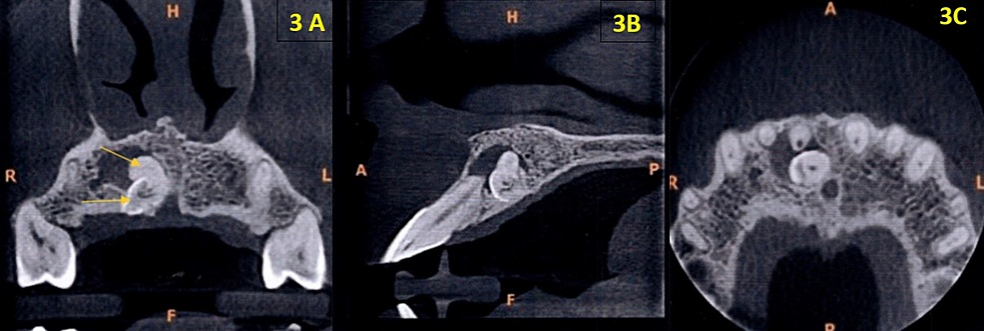
(A) Coronal, (B) sagittal, and (C) axial sections of CBCT CBCT, cone-beam computed tomography

Coronal section of a cone-beam computed tomography (CBCT) image revealed the presence of two well-defined hyperdense masses, one measuring approximately 1 cm in diameter with a peripheral density resembling the enamel of the adjacent teeth and another measuring approximately 1.5 cm in diameter and enclosed in a capsule near the periapical region of the anterior maxillary teeth 21 (Figure 3A). The sagittal section of the CBCT image revealed the presence of two hyperdense masses measuring approximately 1 cm in diameter, one showing a peripheral rim of radiopacity resembling the density of the enamel with a central radiolucent area and another radiopaque mass measuring 3 mm with a central radiolucent canal (Figure 3B). The axial section of the CBCT image revealed the presence of a tooth-like radiopacity with a central canal resembling the morphology of the root canal in a labial location to maxillary anterior tooth #8 with a peripheral rim of radiolucency (Figure 3C).

**Figure 4:**
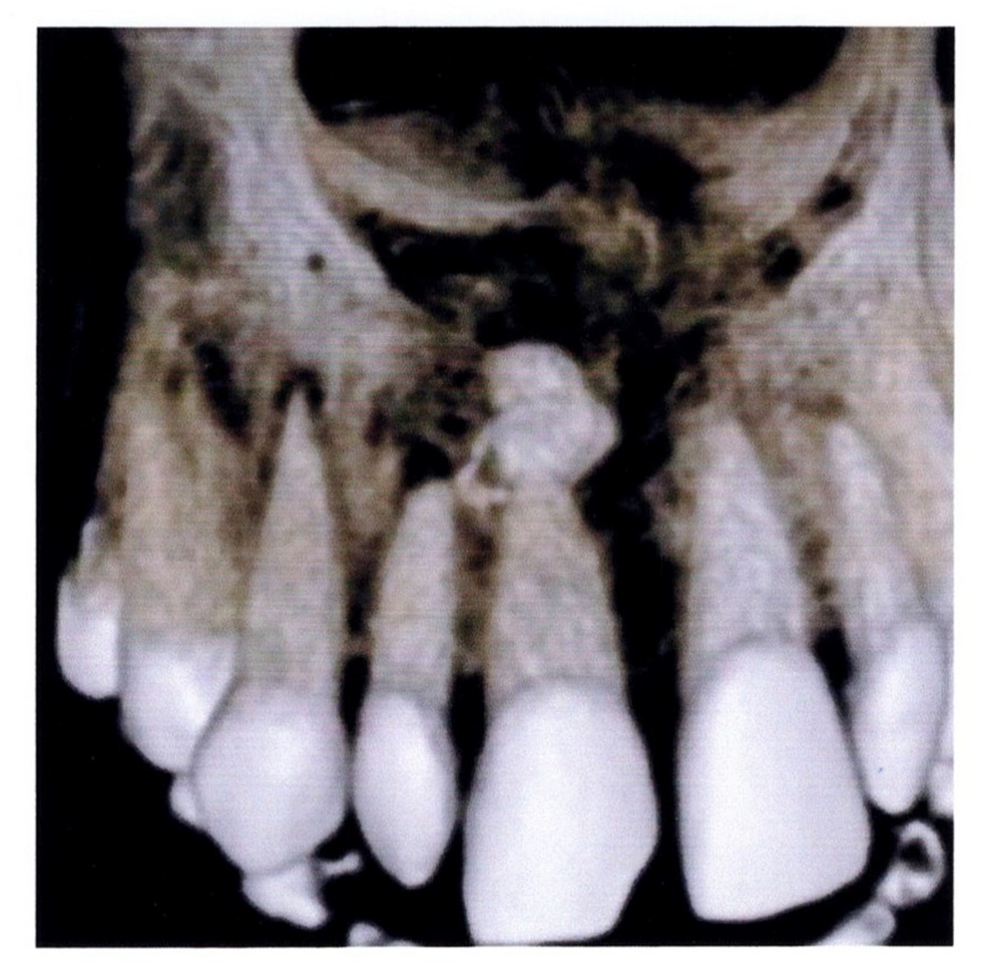
3D-reconstructed CBCT image revealing a radiopaque mass obscuring the root of the broken right maxillary incisor tooth CBCT, cone-beam computed tomography

Three-dimensional reconstructed CBCT image revealed the presence of a well-defined radiopaque mass measuring approximately 1 cm in diameter in the periapical region of the tip of the root, obscuring the root outline (Figure 4).

Treatment

**Figure 5:**
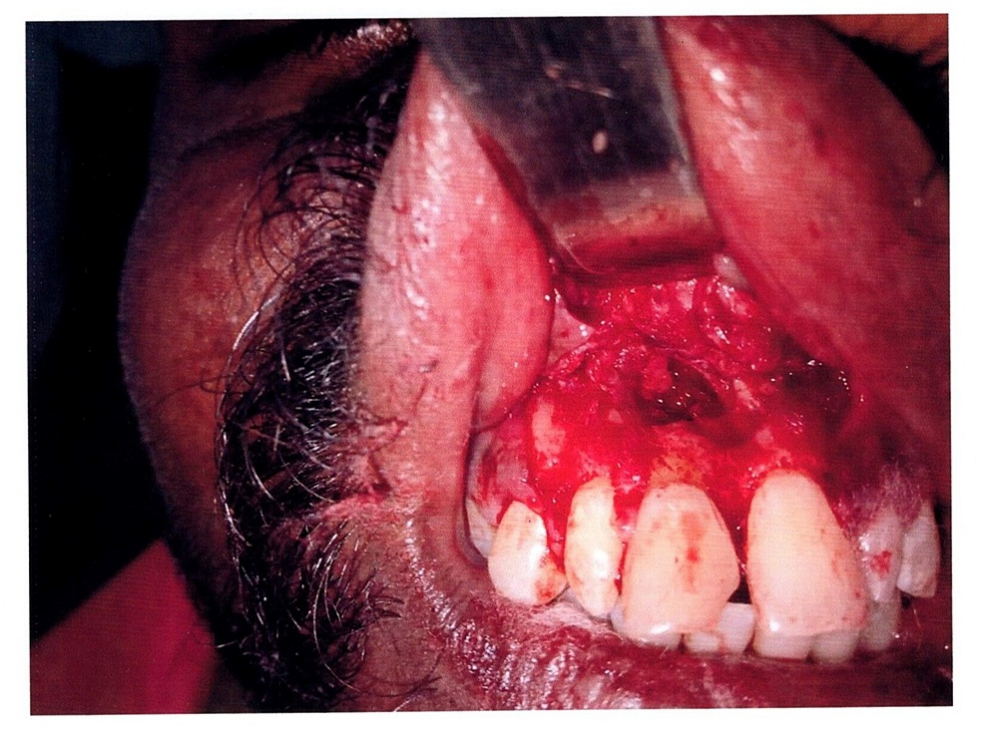
Surgical exploration revealing a radiopaque mass in the periapical root region of the broken right maxillary central incisor tooth

Two vertical releasing incisions were placed in the interdental papilla region in the labial aspect of 12 and another in 22, and the mucoperiosteal flap was elevated. Surgical exploration by elevation of the mucoperiosteal flap revealed the presence of discrete calcareous masses in the apex of the periapical region of the non-vital teeth (Figure 5).

**Figure 6:**
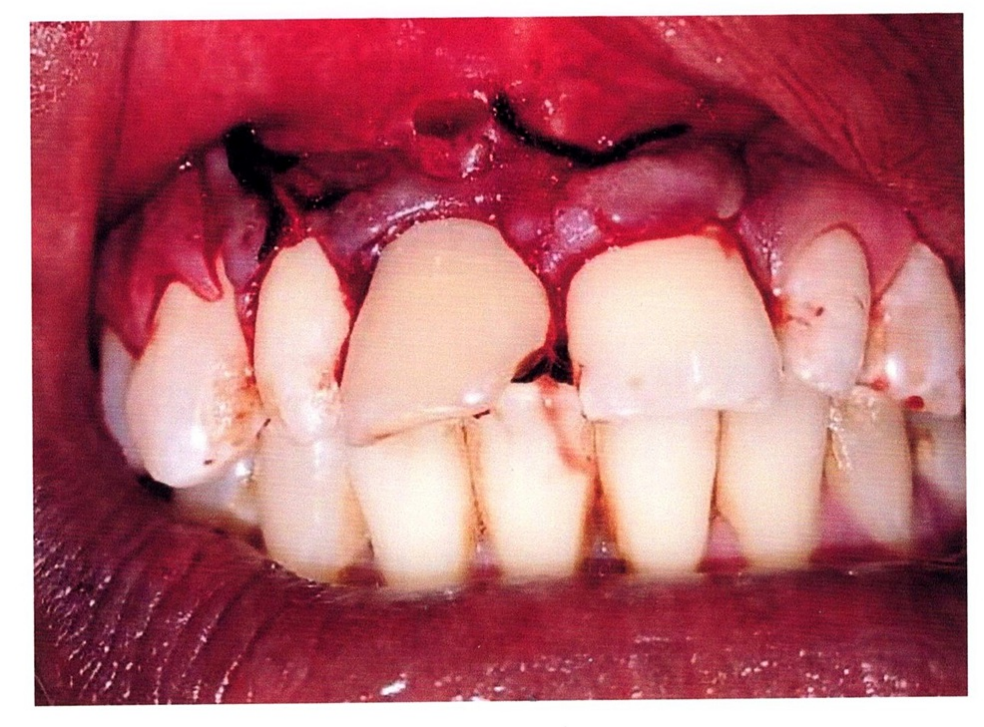
Postoperative intraoral photograph

The calcareous mass was removed, and the area was sutured with a 3-0 black silk suture (Figure 6).

**Figure 7:**
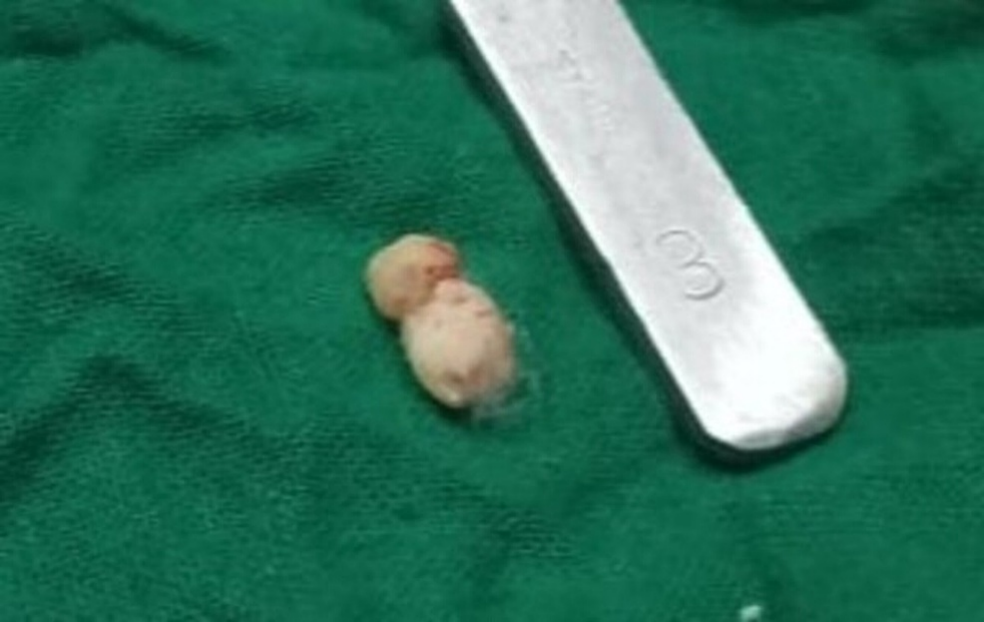
Excised odontoma

The two removed discrete calcareous masses from the periapical region of the non-vital tooth morphologically resembled the crown of the maxillary premolar tooth (Figure 7).

**Figure 8:**
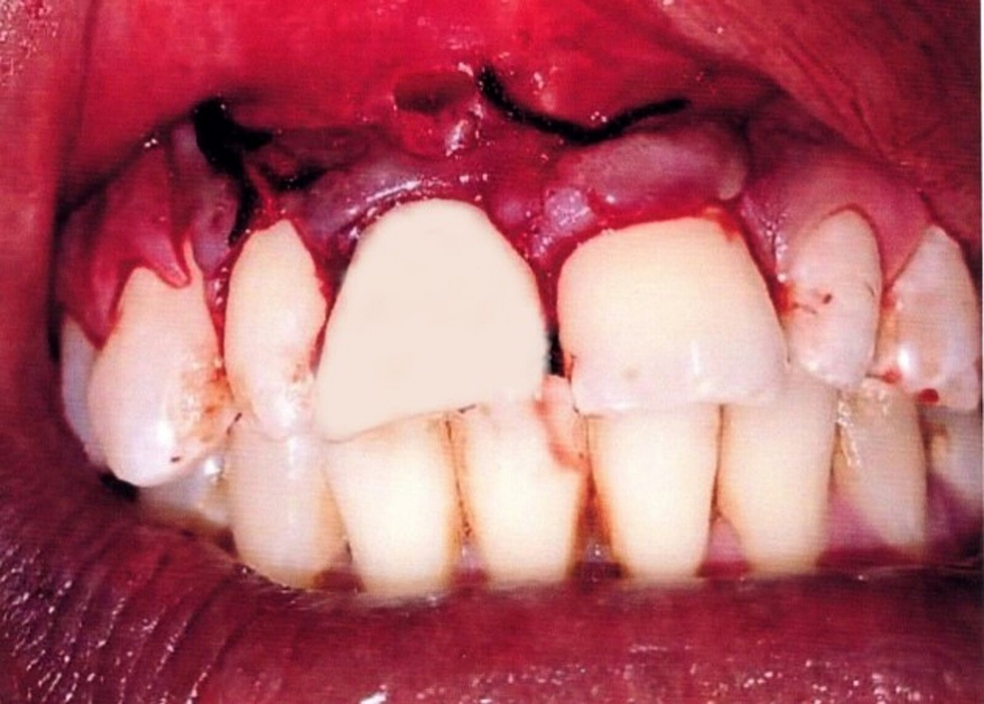
Universal composite anterior restoration done on broken #8

The class IV fracture in #8 was restored with 3M-Filtek Z350 XT Universal Composite restorative material (3M, St. Paul, MN) (Figure 8).

**Figure 9:**
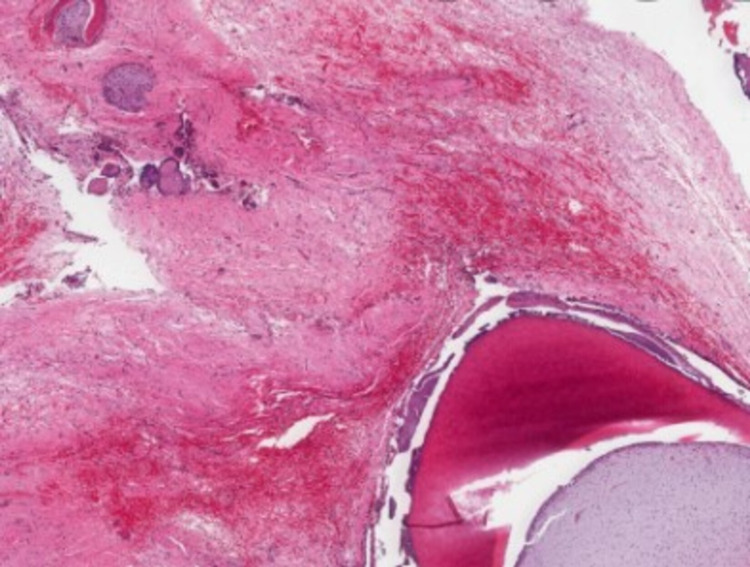
Histopathological photomicrograph (x5) revealing uniform dentin and remnants of pulp tissue

The histopathological section revealed the presence of uniformly arranged dentinal tubule structures with remnants of pulp tissue in a few areas (Figure 9).

Based on the clinical, radiological, and histopathological findings, a final diagnosis of a compound odontoma is made.

## Discussion

Pierre Paul Broca first coined the term “odontoma” in 1866 and defined it as a tumor formed by the overgrowth of complete dental tissue [1,2]. Odontomas are non-neoplastic, benign, hamartomatous lesions occurring in the oral cavity and are typically discovered on routine radiographic examinations. Albrecht, in 1904, coined the term “hamartoma” derived from the Greek word “hamartion,” meaning a bodily defect, or “Hamartanein,” meaning “to make an error.” Odontomas are considered hamartomas due to developmental anomalies resulting from the growth of completely differentiated epithelial and mesenchymal cells that give rise to functional ameloblast and odontoblast. The limited and sluggish growth potential of this lesion distinguishes it from other odontogenic neoplasms, and this is the major reason for considering an odontoma to be a hamartoma or an odontogenic hamartoma. The most salient feature of hamartomas is that they cease growing at some point in their course and do not infiltrate into surrounding tissues. The clinical signs associated with odontoma are unerupted or impacted teeth, presence of retained deciduous teeth or pain, swelling in the vicinity of the affected teeth, or, rarely, cortical expansion or paresthesia of the lip. The etiology of an odontoma is unknown. Some authors suggest a relationship of trauma to underlying primary dentition, underlying inflammatory process, odontoblastic hyperreactivity, disordered odontogenesis, a mutation in the gene during the formation of the tooth, the persistence of cells of the dental lamina, and hereditary anomalies such as Hermann syndrome and Gardner syndrome.

Odontomas are incidentally discovered by routine radiographic examinations at any age in any location in the maxillary or mandibular dental arch. Odontomas rarely erupt into the oral cavity as they do not have roots. However, in older age, odontomas often erupt. The resorption of the alveolar process plays a significant role, and reactive growth of the capsule lining the odontoma and underlying inflammatory process also contributes to this. The rare occurrence of an erupted odontoma is probably due to bone remodeling by the activity of proteases within the dental follicle, secreted by reduced enamel epithelium, which contributes to the eruptive forces in the odontoma that can be detected only in immunohistochemical studies.

In 1946, Thoma and Goldman classified odontoma as follows: geminated composite odontoma, in which two or more or less well-developed teeth fused together; compound composite odontomas, which bear a resemblance to multiple rudimentary teeth-like structures; and complex composite odontomas, in which small, calcified structures bear no resemblance to the tooth.

The crown or root portion of the tooth shows a marked enlargement in dilated odontomas. An odontoma that is normally encapsulated by fibrous connective tissue capsule is called cystic odontomas [3].

Satish et al. classified compound odontoma as follows: denticulo type, which is composed of two or more separate structures having a crown and root, resembling tooth; particulate type, bearing no resemblance to the tooth; and denticulo-particulate type, which is commonly situated in the maxillary incisor-canine region but also located in rare areas near the floor of maxillary sinuses mimicking clinical features of chronic maxillary sinusitis, subcondylar region, ramus of the mandible, mental foramen, midpalatal part, and middle ear [4].

Compound odontomas are hamartomatous malformations in which all dental tissues are represented in a more orderly pattern containing variable amounts of cementum, dentin, and pulp tissue [4]. Compound odontomas generally appear as solitary radiopaque masses accidentally discovered by routine radiographic examinations. In the anterior maxilla, a common site of occurrence is supernumerary teeth, especially mesiodens, which can clinically appear as small conical teeth between maxillary permanent central incisors or impacted or inverted within the maxillary alveolar bone labially or lingually between the two permanent maxillary central incisors.

Compound odontomas consist of discrete, small tooth-like structures usually encapsulated and located in the incisor-cuspid region of the anterior maxilla. Compound odontomas show an irregular radiopaque structure with variations in contour and size, each corresponding to the so-called denticles, and a characteristic radiolucent halo surrounding the radiopacity is usually present that corresponds to the connective tissue capsule surrounding the odontoma. Odontomas can be mistaken for mesiodens but can be differentiated by the lack of radiolucency representing the pulp canal in odontomas. It is therefore important to carefully examine such odontomas by radiographic examination.

Marra et al. stated that odontomas can cause transmigration or deflection of underlying permanent teeth [5]. The most frequently reported location is the incisor-canine area of the maxillary alveolar bone (67%), followed by the mandibular anterior alveolar bone and mandibular posterior region of the mandible (33%). Gupta and Das reported an unusual size of an odontoma invading the orbital floor causing transient diplopia and maxillary sinus resulting in symptoms of chronic maxillary sinusitis [6].

Compound odontomas can also occur in gingival soft tissues. Extragnathic odontoma was first reported by McClure in 1946. Odontoma was also reported in other sites such as the middle ear and tympanum [7]. The literature reviews on cases of odontoma in described in Table 1 [8-11].

**Table 1:** Literature review on cases of odontoma

Year	Author	Age/sex	Clinical findings
2004	Sun et al. [8]	32-year-old male	Odontoma in the middle ear was the cause of ipsilateral and sensorineural hearing loss and was treated by bilateral contralateral routing with the help of a hearing aid
2008	Amailuk and Grober [9]	15-year-old male	Odontoma associated with dilaceration of the left maxillary lateral incisor region
2008	Das et al. [10]	11 year-old male	Compound odontoma in the maxillary incisor region
2010	Gokkulakrishnan et al. [11]	65-year-old female	Odontoma in the right mandibular premolar region excised by sectioning

Clinical significance

Odontomas are asymptomatic and can occur at any age in any areas of the maxillary or mandibular arch and are accidentally discovered by routine radiographic examination. They can lead to buccal, superficial temporal, or submassetric space infection, leading to a diagnostic dilemma [12]. Odontomas need surgical removal if it causes uneruption or deflection of the teeth, devitalization of the tooth that is near its vicinity, or swelling near the affected tooth due to infection. Odontomas do not cause infection themselves but are associated with infections such as an abscess or can undergo cystic degeneration such as the occurrence of dentigerous cysts [12].

Similar to teeth, odontoma does not develop once fully calcified, but they may get exposed to the oral cavity resulting in swelling and pain due to the increased pressure on the adjacent bone resulting in the resorption and thinning of the adjacent bone [13]. Surgical excision of odontomas has a good prognosis [14].

Previous literature reviews reported cases in the apical region of the primary or deciduous tooth. Gill and Yadav have described odontomas in the periapical region of an erupted mandibular primary incisor tooth [14]. Oliveira et al. stated that compound odontoma can occur in calcifying odontogenic cyst resembling a tooth germ [15]. Odontomas are odontogenic tumors with less aggressive behavior [16]. Odontoma rarely erupts in the oral cavity and leads to impaction of underlying tooth [17]. Odontoma can occur in dentigerous cyst [18]. Odontoma in the mandible can lead to neuralgic pain or trigeminal neuralgia involving the mandibular division of the trigeminal nerve [19]. Odontoma can occur in ameloblastic fibro-odontoma and clinically causes gingival enlargement [20].

## Conclusions

Our study described odontoma in an unusual location near the apical region of the broken, discolored maxillary permanent central incisor tooth. Local trauma is the contribution factor for such odontoma. Odontoma can occur anywhere in the toothbearing alveolar bone and also in the mastoid region, orbital floor, or floor of the maxillary sinus. Odontoma can also erupt in the oral cavity and lead to impaction of underlying tooth. The clinical signs and symptoms vary with the location of the odontoma. Odontoma can mimic clinical symptoms of maxillary sinus, if it lies in close proximity involving the floor of the maxillary sinus, or transient diplopia, if it invades the orbital floor. Odontomas themselves do not cause infections but are associated with space infections such as those in the buccal, superficial temporal, and submasseteric spaces. Odontomas are incidentally detected in dentigerous cyst and are also associated with ameloblastic fibro-odontomas masquerading as enlargement of the gingiva. The diagnosis of such odontomas is challenging to the dentist and requires detailed history taking and clinical and radiographic examinations. Odontomas are clinically associated with retained deciduous tooth, transmigration or deflection of permanent teeth, impacted tooth, or tooth anomalies such as fusion and dilaceration. Odontomas in the mandible can also cause trigeminal neuralgia, an electric-shock like neuralgic pain along the mandibular division of the trigeminal nerve. Odontomas are incidentally discovered on routine radiographic examination or when a patient complaints of a broken or missing teeth for aesthetic and prosthetic rehabilitation, respectively. Hence, we conclude that a careful history taking and clinical and radiological examinations are essential in the diagnosis of odontoma, which helps in preventing any diagnostic dilemmas for proper treatment.

## References

[1] Rana V, Srivastava N, Kaushik N, Sharma V, Panthri P, Niranjan MM (2019). Compound odontome: a case report. Int J Clin Pediatr Dent.

[2] Silva DR, Shahinian AL (2022). Odontoma malformation and disturbances of eruption subsequent to traumatic dental injuries: a literature review and a case report [Online ahead of print]. Dent Traumatol.

[3] Isola G, Cicciù M, Fiorillo L, Matarese G (2017). Association between odontoma and impacted teeth. J Craniofac Surg.

[4] Satish V, Prabhadevi MC, Sharma R (2011). Odontome: a brief overview. Int J Clin Pediatr Dent.

[5] Marra PM, Nucci L, Itro A, Santoro R, Marra A, Perillo L, Grassia V (2021). Prevalence of retained/transmigrated permanent and persistence of primary teeth associated with odontomas in young children. Eur J Paediatr Dent.

[6] Gupta M, Das D (2015). Extensive complex odontoma in the maxillary sinus pushing 3rd molar near the orbital floor causing transient diplopia and chronic sinusitis: a rare presentation and surgical management. J Maxillofac Oral Surg.

[7] da Silva Rocha OK, da Silva Barros CC, da Silva LA, de Souza Júnior EF, de Morais HH, da Costa Miguel MC (2020). Peripheral compound odontoma: a rare case report and literature review. J Cutan Pathol.

[8] Sun JJ, Ford LC, Rasgon BM, Lewis BI (2004). Odontoma of the middle ear: case report with 25-year follow-up. Arch Otolaryngol Head Neck Surg.

[9] Amailuk P, Grubor D (2008). Erupted compound odontoma: case report of a 15-year-old Sudanese boy with a history of traditional dental mutilation. Br Dent J.

[10] Das UM, Viswanath D, Azher U (2009). A compound composite odontoma associated with unerupted permanent incisor: a case report. Int J Clin Pediatr Dent.

[11] Gokkulakrishnan Gokkulakrishnan, Singh S, Singh M, Singh KT (2010). A rare case of odontome in a 65-year-old lady. Natl J Maxillofac Surg.

[12] Shetty L, Gangwani K, Kulkarni D, Londhe U (2018). Odontome, cyst, impacted tooth, and space infection in a single patient: all-in-one diagnostic dilemma. Ann Maxillofac Surg.

[13] Perumal CJ, Mohamed A, Singh A, Noffke CE (2011). Erratum to: Sequestrating giant complex odontoma: a case report and review of the literature. J Maxillofac Oral Surg.

[14] Gill NC, Yadav R (2014). A rare case of complex odontoma associated with the root of an erupted mandibular primary incisor. Indian J Oral Sci.

[15] Oliveira EM, Santana LA, Silva ER, Souza LN (2021). A calcifying odontogenic cyst associated with compound odontoma mimicking a tooth germ. Case Rep Dent.

[16] Sánchez-Romero C, Paes de Almeida O, Bologna-Molina R (2021). Mixed odontogenic tumors: a review of the clinicopathological and molecular features and changes in the WHO classification. World J Clin Oncol.

[17] Marya A, Venugopal A (2021). Impaction caused by a rare erupted peripheral compound odontoma. Clin Case Rep.

[18] Kim KS, Lee HG, Hwang JH, Lee SY (2019). Incidentally detected odontoma within a dentigerous cyst. Arch Craniofac Surg.

[19] Stadnicki J (1952). Pripad neuralgie III. vetve trojklaného nervu způsobené odontomem v dolni celisti [Case of neuralgia of the third branch of the trigeminal nerve due to odontoma of the mandible]. Cesk Stomatol.

[20] Nandini DB, Reddy PB, Singh WR, Singh KS (2021). Ameloblastic fibro-odontoma or complex odontoma masquerading as gingival enlargement. J Indian Soc Periodontol.

